# Ten Simple Rules for Getting Grants

**DOI:** 10.1371/journal.pcbi.0020012

**Published:** 2006-02-24

**Authors:** Philip E Bourne, Leo M Chalupa

This piece follows an earlier Editorial, “Ten Simple Rules for Getting Published” [1], which has generated significant interest, is well read, and continues to generate a variety of positive comments. That Editorial was aimed at students in the early stages of a life of scientific paper writing. This interest has prompted us to try to help scientists in making the next academic career step—becoming a young principal investigator. Leo Chalupa has joined us in putting together ten simple rules for getting grants, based on our many collective years of writing both successful and unsuccessful grants. While our grant writing efforts have been aimed mainly at United States government funding agencies, we believe the rules presented here are generic, transcending funding institutions and national boundaries.

At the present time, US funding is frequently below 10% for a given grant program. Today, more than ever, we need all the help we can get in writing successful grant proposals. We hope you find these rules useful in reaching your research career goals.

## Rule 1: Be Novel, but Not Too Novel

Good science begins with new and fresh ideas. The grant writing process should be a pleasure (no, we are not kidding), for it allows you to articulate those ideas to peers who have to read your grants but not necessarily your papers. Look at grant writing as an opportunity to have an impact. Feel passionate about what you are writing—if you are not passionate about the work, it is probably not a good grant and is unlikely to get funded. “Me-too” science will not get funded when funding levels are low. On the other hand, science that is too speculative will not be supported either, particularly when funds are tight—sad but true.

## Rule 2: Include the Appropriate Background and Preliminary Data as Required

You need to convince reviewers that the work you propose needs to be done and that you are the best person to do it. Different granting programs require differing amounts of preliminary data. For certain programs, it can be said that the work must be essentially done before the grant is awarded, and that the funds are then used for the next phase of the research program. There is some truth in this. So where appropriate, do provide some tantalizing preliminary result, making sure to tell the reviewers what these results imply with respect to the specific aims of your proposal. In formulating the motivation for your proposal, make sure to cite all relevant work—there is nothing worse than not appropriately citing the work of a reviewer! Finally, convince the reviewer that you have the technical and scientific background to perform the work as proposed.

## Rule 3: Find the Appropriate Funding Mechanism, Read the Associated Request for Applications Very Carefully, and Respond Specifically to the Request

Most funding organizations have specific staff to assist in finding funding opportunities, and most funding agencies have components of their Web sites designed to help investigators find the appropriate programs. Remember, programs want to give away money—the jobs of the program's staff depend on it. The program staff can help you identify the best opportunities. If your grant does not fit a particular program, save your time and energy, and apply elsewhere, where there is a better programmatic fit.

## Rule 4: Follow the Guidelines for Submission Very Carefully and Comply

Many funding bodies will immediately triage grants that do not comply with the guidelines—it saves the program time and money. This extends to all the onerous supporting material—budget justification, bibliographies, etc. Get them right and keep them updated for future applications. Even if it goes to review, an inappropriately formulated application may aggravate the reviewers, and will have a negative impact even if the science is sound. Length and format are the most frequent offenders.

## Rule 5: Obey the Three Cs—Concise, Clear, and Complete

The grant does not have to fill the allotted page count. Your goal should be to provide a complete reckoning of what is to be done, as briefly as possible. Do not rely on supplements (which may not be allowed) or on Web sites (review may be actively discouraged since it has the potential to compromise anonymity). Specify the scope up-front and make sure it is realistic with respect to the funds requested. A common temptation for inexperienced grant writers is to propose to do too much. Such applications are usually judged as overly ambitious and consequently poorly rated.

## Rule 6: Remember, Reviewers Are People, Too

Typically, reviewers will have a large number of grants to review in a short period. They will easily lose concentration and miss key points of your proposal if these are buried in an overly lengthy or difficult-to-read document. Also, more than likely, not all the reviewers will be experts in your discipline. It is a skill to capture the interest of experts and nonexperts alike. Develop that skill. Unlike a paper, a grant provides more opportunity to apply literary skills. Historical perspectives, human interest, and humor can all be used judiciously in grants to good effect. Use formatting tricks (without disobeying rule 4), for example, underlining, bolding, etc., and restate your key points as appropriate. Each section can start with a summary of the key points.

## Rule 7: Timing and Internal Review Are Important

Give yourself the appropriate lead time. We all have different approaches to deadlines. Ideally, you should complete a draft, leave sufficient time to get feedback from colleagues, and then look at the grant again yourself with a fresh eye. Having a spectrum of scientific colleagues who are similar to the likely reviewer pool critique your grant is very valuable.

## Rule 8: Know Your Grant Administrator at the Institution Funding Your Grant

At the end of the day, this person is your best advocate. How well you understand each other can make a difference. Many grant administrators have some measure (limited to complete) discretionary control over what they fund. The more they know and understand you and your work, the better your chances of success. Do not rely just on E-mail to get to know the grant administrator. Do not be intimidated. Talk to them on the telephone and at meetings where possible—they want to help.

## Rule 9: Become a Grant Reviewer Early in Your Career

Being on review panels will help you write better grants. Understanding why grants get triaged before complete review, how a panel reacts to a grant, what the discretionary role of program officers is, and what the role of oversight councils is provide valuable lessons for writing successful grants of your own and for giving others advice about this process.

## Rule 10: Accept Rejection and Deal with It Appropriately

Rejection is inevitable, even for very good grants when funding levels are low. Learn to live with rejection and to respond appropriately. Do not be defensive; address each criticism head on and respond with facts and not emotional arguments. When resubmission is necessary, make it very clear to the reviewer that you understand what was wrong the first time. Indicate precisely how you have fixed the problems. In the resubmitted application, never argue with the validity of the prior review. If the grant was close to being funded the first time around, remind the reviewers of that fact by including the previous score if appropriate, and make it crystal clear why this version is much improved.

There are no previously unrevealed secrets to grant writing presented here. Rather, it is a concise picture intended to help our early career readers take the next step. If you feel like you need more detail, take a look at Kraicer's article [2]. Good luck on getting those grants.

## References

[pcbi-0020012-b001] Bourne PE (2005). Ten simple rules for getting published. PLoS Comput Biol.

[pcbi-0020012-b002] Kraicer J (1997). The art of grantmanship.

